# Factors associated with self-reported number of teeth in a large national cohort of Thai adults

**DOI:** 10.1186/1472-6831-11-31

**Published:** 2011-11-24

**Authors:** Vasoontara Yiengprugsawan, Tewarit Somkotra, Matthew Kelly, Sam-ang Seubsman, Adrian C Sleigh

**Affiliations:** 1 National Centre for Epidemiology and Population Health, The Australian National University, Canberra, Australia; 2 Department of Community Dentistry, Faculty of Dentistry, Chulalongkorn University, Bangkok, Thailand; 3 School of Human Ecology, Sukhothai Thammathirat Open University, Nonthaburi, Thailand

## Abstract

**Background:**

Oral health in later life results from individual's lifelong accumulation of experiences at the personal, community and societal levels. There is little information relating the oral health outcomes to risk factors in Asian middle-income settings such as Thailand today.

**Methods:**

Data derived from a cohort of 87,134 adults enrolled in Sukhothai Thammathirat Open University who completed self-administered questionnaires in 2005. Cohort members are aged between 15 and 87 years and resided throughout Thailand. This is a large study of self-reported number of teeth among Thai adults. Bivariate and multivariate logistic regressions were used to analyse factors associated with self-reported number of teeth.

**Results:**

After adjusting for covariates, being female (OR = 1.28), older age (OR = 10.6), having low income (OR = 1.45), having lower education (OR = 1.33), and being a lifetime urban resident (OR = 1.37) were statistically associated (p < 0.0001) with having less than 20 teeth. In addition, daily soft drink consumptions (OR = 1.41), current regular smoking (OR = 1.39), a history of not being breastfed as a child (OR = 1.34), and mother's lack of education (OR = 1.20) contributed significantly to self-reported number of teeth in fully adjusted analyses.

**Conclusions:**

This study addresses the gap in knowledge on factors associated with self-reported number of teeth. The promotion of healthy childhoods and adult lifestyles are important public health interventions to increase tooth retention in middle and older age.

## Background

Socio-behavioral and environmental factors have been shown to have epidemiologically important roles in oral health [1-5]. Poor living conditions and limited access to oral health services are important risk factors. In addition, unhealthy lifestyles are substantial determinants of oral health, including poor diet, inadequate oral hygiene, tobacco smoking and alcohol drinking [6,7].

Oral health in later life results from an individual's lifelong accumulation of advantageous and disadvantageous determinant experiences at the personal, community and societal levels. These experiences differ according to gender, race, and various socioeconomic factors such as education, income, and occupation [8]. Several birth cohort studies have shown that socioeconomic inequalities in childhood are associated with differing levels of oral health outcomes in adulthood [9-11]. Good oral health is significantly linked with well-being in old age and the importance of oral health among elderly populations has been demonstrated in various settings [12-14]. Therefore, understanding the epidemiology of oral diseases and their socioeconomic patterns across the life course is crucial for determining optimal times for interventions to limit oral disease burden in populations.

According to the Thai National Oral Examination Surveys of 1996, 2001, and 2006, the proportion of the adult population with more than 20 remaining teeth is increasing, which are encouraging for Thailand, but there are still substantial disparities across the country (91% vs 92% vs 96% respectively) [15]. The epidemiology of tooth retention and oral health in Asia has mostly been studied in high income countries, especially in Japan and South Korea [16-18]. But there is very little information relating the oral health outcomes to their potential determinants in middle and lower income settings.

To address this knowledge gap on determinants of oral health status, we collected tooth retention data along with detailed information on socio-demographic characteristics, health-risk behaviors and lifecourse history from a large cohort of adults residing throughout Thailand. We have been studying this cohort since 2005 to characterize the health-risk transition as the Thai population moves on from a developing country to a pattern of chronic non-communicable diseases [19-22]. Here we report our analysis of factors associated with self-reported number of teeth among our Thai cohort. This information should help to develop strategies and measures to improve oral health and related wellbeing in the Thai population.

## Methods

### Study population and data collection

In 2005, baseline questionnaires were mailed out to about 200,000 adult students enrolled at Sukhothai Thammathirat Open University (STOU) and 87,134 (44%) aged 15 to 87 years responded. Details on cohort enrolment and overall methodology have been reported elsewhere [22]. Responding students were similar to other STOU students for age, sex, marital status, income, courses of study and geographical location. Furthermore, the cohort participants were generally similar to the population of Thailand, especially in the 20-39 years age group, for sex ratio, geographical location, and socioeconomic status measured by median income [22]. Participation in the study was voluntary and study leaders reassured participants that their personal responses were confidential. Cohort members were motivated by being fully informed about the purposes of the Thai Health-Risk Transition study and that they could contribute to public health knowledge in Thailand. A periodic newsletter provides information back to cohort members on study progress and follow-up continues [23].

The 2005 20-page baseline questionnaire covered a wide range of topics including demographic, socioeconomic and geographic attributes, health status including self-reported number of teeth, health service use, risk behaviors, injuries, dietary intake, physical activities, and family background. The questionnaire was developed by a multi-disciplinary team of experts in both Thailand and Australia. As far as possible we used standardized validated measures, including those used by the Thai National Statistical Office (e.g. income categories, geographical location, and household assets). There were many iterations and extensive pretesting of back translated Thai versions to ensure face and content validity. Pilot testing preceded the final version.

Here we analyse self-reported number of teeth and relate that to sex; age (3 groups: 15-29 years, 30-49 years, and 50 years and above), monthly income in Thai Baht (≤3000; 3001-7000; 7001-10000; and >10000; 40 Baht~$1US in 2005), education (highschool, post high school diploma/certificate, university or higher), and household assets (later categorized by total replacement value in Thai Baht into three groups: <30,000; 30,001-60,000; and >60,000). The household assets included general domestic items such as a microwave oven, electric fan, air conditioner, computer, radio, video/vcd recorder, washing machine, water heater, and telephone).

As well, we determined lifecourse urbanization based on geographic residence now (as an adult member of the cohort) and when aged 12 years--creating the following urbanization categories ('lifetime rural residents'; 'rural-urban migrants'; and 'lifetime urban residents'). The small number of cohort members (4%) who were categorized as 'urban-rural migrants' were excluded from the analysis so we could concentrate on the main categories that characterized the Thai population today.

### Self-reported number of teeth

Outcome was assessed by the question: "Adults can have up to 32 natural teeth. How many of your own teeth do you have?" Self-reported number of teeth was reported in 4 categories: 'none', '1-5', '6-19', and '20+', and was then dichotomized into <20 or ≥20.

### Other covariates

Health-risk behavior variables included current regular tobacco smoking (never smoker, ex-smoker, regular smoker), alcohol drinking (regular or not), and soft drink intake (at least daily, or not). Childhood covariates were history of being breastfed as a child recalled by inquiring from relatives (yes, no, or do not know), father's education, and mother's education.

### Data processing and statistical analysis

Data scanning and editing were done using Thai Scandevet, SQL and SPSS software. For analysis we used Stata version 10. Individuals with missing data for analyses presented here were excluded so totals vary a little according to the information available. Missing data usually involved 1% or less of observations, however, our results were stable given the large size of our dataset. Bivariate analysis was performed followed by stepwise multivariate logistic regression (with p set at < 0.05). Before multivariate analyses were conducted, independent variables were tested for collinearity; no correlation coefficient exceeded 0.52. Notably, socioeconomic variables (income, education, and household assets) had correlation coefficients lower than 0.34.

### Ethical considerations

Ethics approval was obtained from Sukhothai Thammathirat Open University Research and Development Institute (protocol 0522/10) and the Australian National University Human Research Ethics Committee (protocol 2004344). Informed written consent was obtained from all participants.

## Results

### Characteristics of the cohort members

There were 87,134 cohort members (Table 1) and 45.3% were males. Three age groups ranged from 15 to 29 years (53.6%), 30 to 49 years (43.9%), 50 years and above (2.5%). There were lower proportions of males among the younger group and lower proportions of females among the older group. Among cohort members, 42.1% were married and the proportion increased with age. Approximately 11% of cohort members reported income less than 3,000 Baht and 33.9% reported income more than 10,000 Baht per month. There was a clear positive trend between age and income. Three groups of lifecourse urbanization were analysed: lifetime rural residents (43.3%); rural moved to urban areas since age 12 years (31.5%); and lifetime urban residents (20.0%).

**Table 1:** Characteristics of cohort members by age groups

		Age groups %		
		
Attributes	N = 87,134	n = 46,727 20-29 yrs	n = 38,257 30-49 yrs	n = 2,150 50 yrs+
**Overall**		53.6	43.9	2.5
Socio-demographic characteristics*				
Males	**45.3**	37.9	52.8	71.9
*Marital status*				
Married	**42.1**	22.7	33.0	77.2
*Income (Baht/month)*				
<3,000	**10.8**	16.1	4.6	4.7
3,001-7,000	**30.1**	41.7	17.3	8.9
7,001-10,000	**22.7**	26.1	19.5	7.3
>10,000	**33.9**	13.3	56.7	76.1
*Education (before enrollment at STOU)*				
Up to high school education	**48.7**	48.8	48.5	50.5
Post high school diploma/certificate	**26.9**	31.3	22.2	16.4
University degree	**24.1**	19.7	29.0	32.6
*Household assets (in Baht)*				
0-30,000	**40.4**	50.0	30.0	20.7
30,001-60,000	**30.5**	29.4	32.1	27.5
>60,000	**28.6**	20.2	37.6	50.8
*Lifecourse urbanization*				
Lifetime rural residents	**43.3**	47.3	39.3	28.2
Rural-urban residents	**31.5**	29.9	33.3	32.6
Lifetime urban residents	**20.0**	18.2	21.0	29.5
**Health behaviors**				
*Smoking*				
Never smoker	**70.1**	77.5	62.5	45.7
Ex smoker	**15.4**	10.3	12.1	10.7
A regular smoker	**9.8**	7.9	20.3	37.2
*Alcohol drinking*				
A regular alcohol drinker	**4.8**	2.9	6.9	7.9
*Soft drink consumption*				
Consume soft drink daily	**7.6**	9.4	5.6	4.4
**Personal history**				
*Breastfed as a child*				
Not breastfed as a child	**7.0**	7.9	6.2	6.1
*Father's education*				
Father no formal education	**5.6**	4.2	6.9	13.4
*Mother's education*				
Mother no formal education	**10.2**	7.5	12.5	26.4

*In some categories totals do not add up to 100% due to missing values.

Regarding health behaviors, 9.8% reported being a current regular smoker, 4.8% reported being a current regular alcohol drinker. Both health risk behaviors increase with age (37.2% regular current smoker among older persons compared to 7.9% among the youngest group). Daily consumption of carbonated beverage was reported by 7.6% of the cohort members, 9.4% among the youngest group compared to 4.4% among the oldest group.

Not being breastfed as a child was reported by 7.0%, generally a little less in older groups. Parental history of a father with lower than primary education was 4.2% among the cohort members aged 15 to 29 years old compared to 13.4% among the cohort members aged 50 years and above. A similar trend was noticed for less than primary education for mothers of younger vs. older cohort members (7.5% vs. 26.4%)

### Self-reported number of teeth

Among cohort members, 3.3% reported having less than 20 teeth (2.4% for 15-29 year-olds vs. 17.2% for those aged 50 years or more). The older group consistently reported less than 20 teeth across all other characteristics included in Table 2 where age was the most prominent factor associated with less than 20 teeth. Those in the lowest income group were more likely to report less than 20 teeth compared to the highest income group and this trend increased with age. Across all age groups, lifetime urban residents were more likely than lifetime rural residents to report less than 20 teeth (overall 4.4% vs 2.9%).

**Table 2:** Self-reported number of teeth by attributes of cohort members

Attributes	Less than 20 teeth (%)			
		
		Age groups		
	**N = 87,134**	**n = 46,727** **20-29 yrs**	**n = 38,257** **30-49 yrs**	**n = 2,150** **50 yrs+**
**Overall**	**3.3**	2.4	3.6	17.2
**Socio-demographic characteristics**				
Males	**3.3**	2.1	3.2	17.9
Females	**3.3**	2.6	4.0	15.2
*Marital status*				
Married	**3.6**	2.0	3.5	16.5
Non married	**3.0**	2.5	3.8	20.0
*Income (Baht/month)*				
<3,000	**3.8**	1.6	3.5	16.7
3,001-7,000	**2.6**	1.8	3.4	15.3
7,001-10,000	**3.1**	1.6	3.5	16.8
>10,000	**3.5**	2.0	3.3	15.3
*Education (before enrollment at STOU)*				
Up to high school education	**3.6**	2.7	3.7	17.9
Post high school diploma/certificate	**3.2**	2.3	4.0	20.7
University degree	**2.9**	1.6	3.2	14.4
*Household assets (Baht)*				
0-30,000	**3.1**	2.1	3.7	15.1
30,001-60,000	**3.3**	2.4	3.6	19.3
>60,000	**3.6**	2.5	3.5	19.6
*Lifecourse urbanization*				
Lifetime rural residents	**2.9**	2.3	3.2	16.0
Rural-urban residents	**4.8**	2.2	2.9	14.3
Lifetime urban residents	**4.4**	2.7	5.0	19.7
**Health behaviors**				
*Smoking*				
Never smoker	**3.0**	2.4	3.5	14.6
Ex smoker	**3.7**	2.0	3.4	17.6
A regular smoker	**4.1**	2.1	4.6	27.7
*Alcohol drinking*				
Not a regular alcohol drinker	**3.2**	2.4	3.6	16.5
A regular alcohol drinker	**4.2**	2.4	4.0	21.1
*Soft drink consumption*				
Not consume soft drink daily	**3.2**	2.3	3.5	17.0
Consume soft drink daily	**4.2**	3.5	5.0	20.2
**Personal history**				
*Breastfed as a child*				
Reported breastfed as a child	**3.1**	2.3	3.4	16.9
Not breastfed as a child	**4.1**	3.2	5.0	17.0
*Father's education*				
Father no formal education	**4.5**	2.5	4.2	21.3
Father primary education and above	**3.2**	2.4	3.6	16.4
*Mother's education*				
Mother no formal education	**4.5**	2.3	4.6	18.0
Mother primary education and above	**3.1**	2.4	3.5	17.0

Self-reported less than 20 teeth was more common among current regular smokers (4.1% vs. 3.0%), current regular alcohol drinkers (4.2% vs. 3.2%), and cohort members who consumed soft drink daily (4.2% vs. 3.2%). Having a history of not being breastfed as a child was associated with less than 20 teeth (4.1% vs 3.1%). Those with father's education of less than primary level were also more likely to have less than 20 teeth (4.5% vs. 3.2%).

### Factors associated with self-reported number of teeth

Bivariate and multivariate results of determinants for self-reported number of teeth are shown in Table 3. After adjusting for all covariates accepted by the stepwise multivariate logistic regression model, being female (OR = 1.28), older age (OR = 10.6), having low income (OR = 1.45), having lower education (OR = 1.33), and being a lifetime urban resident (OR = 1.37) were all associated with less than 20 teeth.

**Table 3:** Factors associated with self report of less than 20 teeth among cohort members

Attributes	Bivariate		Stepwise multivariate*	
	
	Odds Ratios	p-value	Odds Ratios	p-value
**Socio-demographic characteristics**				
Males	1			
Females	**1.19**	0.000	**1.28**	0.000
15-29 years	1			
30-49 years	**1.56**	0.000	**1.79**	0.000
50+ years	**9.05**	0.000	**10.6**	0.000
Married	1			
Non married	1.14	0.002		
*Income (Baht/month)*				
<3,000	**1.48**	0.000	**1.45**	0.000
3,001-7,000	**1.25**	0.004	**1.26**	0.000
7,001-10,000	0.96	0.426		
>10,000	1			
*Education (before enrollment at STOU)*				
Up to high school education	**1.38**	0.000	**1.33**	0.000
Post high school diploma/certificate	**1.32**	0.000	**1.24**	0.000
University degree	1			
*Household assets (Baht)*				
0-30,000	1.09	0.215		
30,001-60,000	1.06	0.071		
>60,000	1			
*Lifecourse urbanization*				
Lifetime rural residents	1			
Rural-urban residents	0.91	0.061		
Lifetime urban residents	**1.34**	0.000	**1.37**	0.000
**Health behaviors**				
*Smoking*				
Never smoker	1			
Ex smoker	**1.17**	0.010		
A regular smoker	**1.46**	0.000	**1.39**	0.000
*Alcohol drinking*				
Not a regular alcohol drinker	1			
A regular alcohol drinker	**1.24**	0.011		
*Soft drink consumption*				
Not consume soft drink daily	1			
Consume soft drink daily	**1.50**	0.000	**1.41**	0.000
**Personal history**				
*Breastfed as a child*				
Reported breastfed as a child	1			
Not breastfed as a child	**1.29**	0.000	**1.34**	0.000
*Parental education*				
Father no formal education	**1.19**	0.015		
Father primary education and above	1			
Mother no formal education	**1.18**	0.003	**1.20**	0.005
Mother primary education and above	1			

*Based on multivariate logistic regression using Stata 10 (see Methods).

In addition, daily soft drink consumption (OR = 1.41) and current regular smoking (OR = 1.39) were also positively associated with less than 20 teeth, but there was no statistically significant association to current alcohol drinking after adjusting in the full model. A history of not being breastfed as a child was statistically associated with less than 20 teeth (OR = 1.34). Mother's education (but not the father's) was associated with less than 20 teeth after adjusting for covariates (OR = 1.20).

## Discussion

To our knowledge, this is the first large scale study that assessed the factors associated with self-reported number of teeth among Thai adults. Key findings of our study were being female, of older age, low income, lower education, and being a lifetime urban resident were all strongly associated with less than 20 teeth. In relation to urban or rural residence, we cannot be sure if the self-reported number of teeth reflects differential access to tooth extraction. Formal access to dental care is better in cities but tooth extraction (for pain relief) is more common in rural areas and amongst lower income.

Our study is consistent with previous studies on the role of health-related behaviors, especially tobacco smoking on self-reported number of teeth. A Japanese study found the odds ratios of having more than eight missing teeth was much higher among current smokers compared to males who had never smoked (OR = 1.67) [24]. An Australian '45 and Up' cohort study based on over 100,000 participants also showed that current smokers had significantly higher odds of tooth loss compared with never smokers (OR = 2.51) [25].

Our findings that consumption of soft drink or carbonated beverage was strongly associated with self-reported number of teeth among our cohort, confirms previous studies [26-28]. Undesirable oral health behaviors adopted in early years are likely to be sustained throughout life and together with cumulative effects of exposure to other risk factors can lead to poor oral health outcomes in later life.

Our study also showed the influence of certain lifecourse factors such as a history of breastfeeding and maternal education on self-reported number of teeth. A study in New Zealand demonstrated that childhood circumstances have a major influence on oral health in adulthood [9,10]. After controlling for childhood oral health, those who were disadvantaged at the age of 5 years had higher levels of dental caries and periodontal disease and were more likely to experience premature tooth loss in adulthood. Hence, the promotion of healthy childhoods is important public health interventions to increase tooth retention in middle and older age.

Thailand has implemented a Universal Coverage Scheme (UCS) for healthcare since 2001 and basic oral healthcare was part of the package [29,30]. However, a recent study based on over 30,000 Thai adults aged 15 and 75 years from nationally representative surveys reported that substantial socioeconomic inequality in self-reported oral health status still persisted after the UCS [31,32]. It should be noted that this representative study was carried out only two years after the UCS reached the whole Thai population, meaning that there may not be sufficient time for measurable improvement on oral health status.

It is important to take into account the possible limitations of our study. For example, our cohort members are university educated and therefore results may not be generalized to the Thai population at large. In addition, there could be some possible recall bias in reporting past behaviors, such as being breastfed as a child. Our data presented here were derived from the cross-sectional baseline questionnaire of our study. Longitudinal data will provide more insight into determinants of self-reported number of teeth.

Although, this study relied on self-reported number of teeth it should be noted that this simple measurement is now used in an increasing number of studies and does capture important information on oral health morbidity [33]. Further studies should emphasise some aspects of positive outcomes of oral health and oral health-related quality of life to fill gaps in information concerning oral health outcomes [34].

## Conclusion

This study addresses the gap in knowledge on factors associated with self-reported number of teeth. Major challenges of the future will be to translate knowledge and experiences of oral health promotion and disease prevention into action programs in Thailand. For instance, dental health should be integrated into public health programs targeting chronic diseases. Sustainable oral health improvements in Thailand require an approach which addresses the underlying determinants of oral health. Many of those determinants have been noted in this study, including sociodemographic risk factors and adverse health behaviours.

## Competing interests

The authors declare that they have no competing interests.

## Authors' contributions

VY and TS conceptualised, analysed and drafted the manuscript. MK provided editorial support. SS and AS designed the project and provided comments. The Thai Cohort Study Team contributed to various stages of the project. All authors read and approved the final manuscript.

## Pre-publication history

The pre-publication history for this paper can be accessed here:

http://www.biomedcentral.com/1472-6831/11/31/prepub
